# New‐onset vitiligo following mRNA‐1273 (Moderna) COVID‐19 vaccination

**DOI:** 10.1002/ccr3.4865

**Published:** 2021-09-26

**Authors:** Joshua Kaminetsky, Donald Rudikoff

**Affiliations:** ^1^ Department of Dermatology Icahn School of Medicine at Mount Sinai New York New York USA; ^2^ Department of Dermatology BronxCare Health System Bronx New York USA

**Keywords:** adverse reaction, COVID‐19, mRNA vaccine, vitiligo

## Abstract

The COVID‐19 mRNA vaccines not only provide remarkable protection but also have been characterized by an overall safe and well‐tolerated side effect profile. Herein, we discuss a rare but manageable cutaneous reaction to COVID vaccination in order to further characterize dermatologic reactions and stress the continued vaccination of eligible patients.

## Graphical abstract

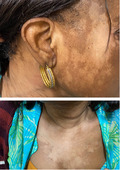

## 1 REPORT OF A CASE

The COVID‐19 mRNA vaccines not only provide remarkable protection but also have been characterized by an overall safe and well‐tolerated side effect profile. Herein, we discuss a rare but manageable cutaneous reaction to COVID vaccination in order to further characterize dermatologic reactions and stress the continued vaccination of eligible patients.

A 61‐year‐old woman presented to our clinic 3 days after her second dose of the Moderna (mRNA‐1273) COVID‐19 vaccine. Several days after her first dose, the patient noted very faint hypopigmented macules on her anterior neck, but she did not seek treatment. After her second dose, these macules progressed in size and degree of hypopigmentation, and she developed new and more widespread macules on other parts of her body.

The patient reported no personal nor family history of vitiligo, autoimmune disease, and no other pigmentary disorders. She had no comorbid health conditions, nor took any medications. No treatment had been attempted prior to the presentation.

**FIGURE 1:**
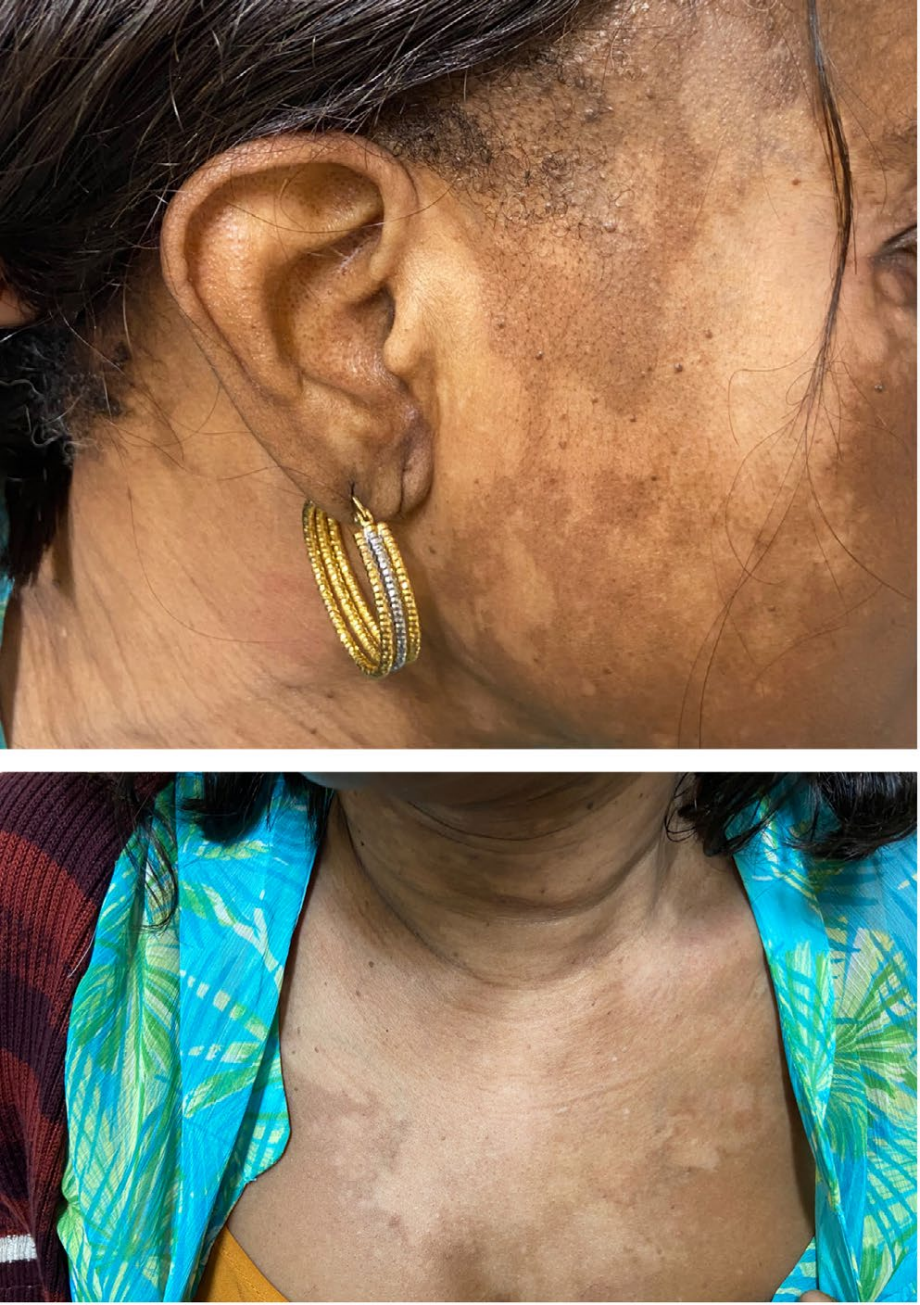
Depigmented patches on the patient's face, neck, and chest

**FIGURE 2:**
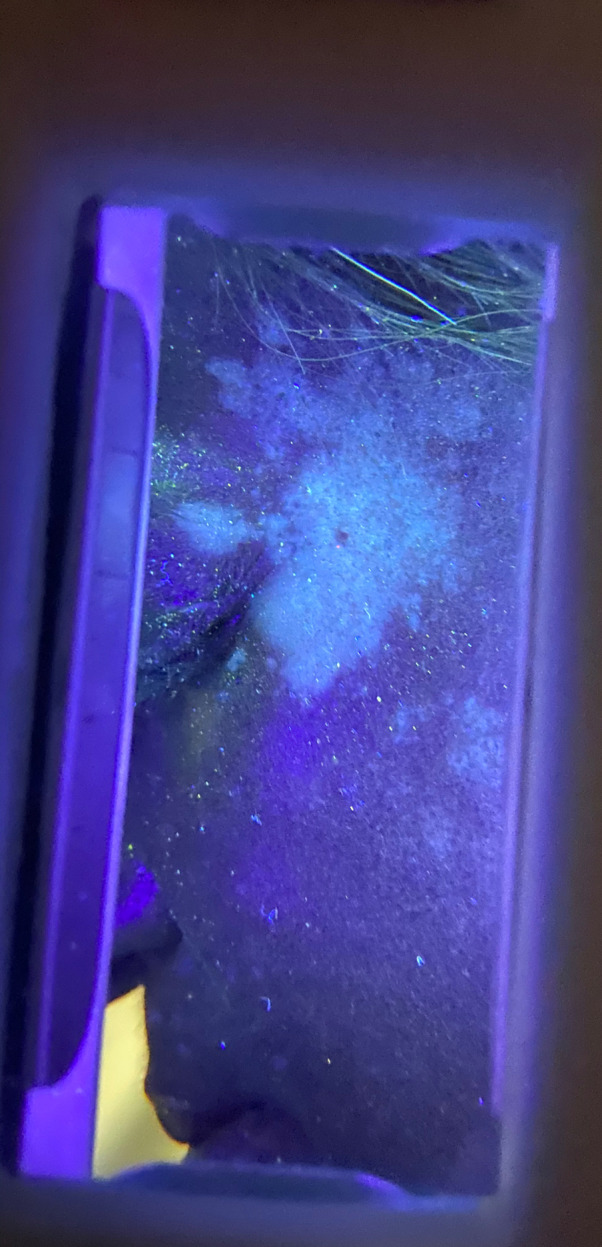
Wood's lamp examination demonstrating accentuation of patches

Examination revealed Fitzpatrick type V skin with widespread depigmented macules on the face, neck, chest, and abdomen (Figure 1). Wood's lamp examination of the lesions demonstrated an accentuated “milky‐white” appearance of the affected lesions, (Figure 2) consistent with a diagnosis of vitiligo. She was prescribed a topical calcineurin inhibitor and started on a phototherapy regimen.

## 2 DISCUSSION

To date, several registry reviews and case series have documented cutaneous vaccine reactions, most commonly local injection reactions, coined “COVID Arm”,^1^ and other reactions that include generalized urticarial or morbilliform eruptions, zoster flares,^2^ and chilblains.

While vitiligo has not been frequently documented, there is one report describing a patient with ulcerative colitis who developed vitiligo several days after administration of the Pfizer vaccine.^3^ To our knowledge, our case is the first case of vitiligo in a patient without a history of autoimmunity, as well as the first case following vaccination with the Moderna vaccine. While no mechanistic explanation has been characterized that can definitively implicate the vaccine as the cause of the vitiligo, the temporal association and lack of a more plausible explanation seem to indicate that vaccine was, at least in part, contributory. There have been other instances of autoimmune phenomena manifesting after COVID‐19 vaccination, such as immune thrombocytopenic purpura,^4^ a phenomenon in which platelets are targeted by the immune system for destruction, much like melanocytes in vitiligo. There is also precedent of autoimmunity following vaccination with other older vaccines, such as MMR, polio, and TdAP. Given the potent immune response generated by the COVID‐19 vaccines, one can speculate that other unintended alterations in a vaccinated individual's immune milieu can occur. Other cells, like melanocytes in the case of vitiligo, may become inadvertent targets of the antibodies and immune cells newly generated and galvanized by the vaccine.

Vitiligo, while not life‐threatening, can pose significant challenges for a patient's psychosocial well‐being, especially in skin of color patients whose disease is more readily noticeable. We present this case to add the mounting literature regarding possible COVID‐19 dermatologic adverse reactions, in order to assist clinicians in counseling their patients regarding the spectrum of possible cutaneous reactions. For perspective, tens of millions of individuals have now been vaccinated and only two reports of vaccine‐induced vitiligo exist to date. The above case notwithstanding, the risk of permanent and serious reactions to vaccination—dermatologic and otherwise—is far outweighed by the benefits conferred by vaccination, both to the vaccinated individual and society at large. We encourage all eligible candidates to continue to receive these remarkably effective and safe vaccines in order to bring a swift and long‐overdue end to this catastrophic pandemic.

## CONFLICTS OF INTEREST

The authors report no conflicts of interest, financial or otherwise.

## AUTHOR CONTRIBUTIONS

Dr. Kaminetsky is the resident physician who evaluated and treated the patient and drafted the manuscript. Dr. Rudikoff is the attending physician who oversaw the care of the patient and edited the manuscript.

## ETHICAL APPROVAL

This manuscript is the author's own original work, which has not been previously published elsewhere. All authors have actively contributed to this work, and all author contributions have been properly credited. All citations have been properly disclosed. The authors report no conflicts of interest, financial or otherwise.

## CONSENT

Informed consent has been obtained from any participant whose features are readily identifiable in any media presented in the manuscript. Any necessary consent has been properly documented, signed, and collected in written format.

## Data Availability

Data sharing is not applicable to this article, as no datasets were generated or analyzed during the current study.
